# When Shortness of Breath Gets Misdiagnosed: Atrial Myxoma Case Report

**DOI:** 10.7759/cureus.34740

**Published:** 2023-02-07

**Authors:** Sahaam Mirza, Tori Tyler, Robert Stauffer

**Affiliations:** 1 Cardiology, Kansas City University of Medicine and Biosciences, Kansas City, USA; 2 Osteopathic Medicine, Kansas City University, Kansas City, USA; 3 Cardiology, Freeman Health System, Joplin, USA

**Keywords:** echocardiogram, left atrium, shortness of breath, cardiac tumor, atrial myxoma

## Abstract

The vague presentation of an atrial myxoma makes accurate diagnosis difficult. This case report reviews the case of a 54-year-old female who presented to the Emergency Department complaining of shortness of breath and back pain. Prior to her Emergency Department evaluation, she had been given a diagnosis with pneumonia. Upon further evaluation, she was diagnosed with a large left atrial myxoma that was surgically removed via median sternotomy. After recovering from her surgery, the patient had a full resolution of her symptoms. Although the treatment for myxomas has a high success rate of full recovery, the difficulty in diagnosis poses problems for patients and can prolong symptoms due to potential misdiagnosis.

## Introduction

Cardiac tumors are rare in nature, as their prevalence has been reported to be 0.001% to 0.030% [1]. The most common cardiac tumor, which accounts for almost half of the cardiac tumors, are myxomas which are benign. Myxoma tumors are suggested to originate from multi-potent mesenchymal cells which have the ability to undergo neural and endothelial differentiation [2]. Literature shows that the exact etiology of this tumor remains unknown. Clinical manifestations of the tumor are dependent on the size and location of the tumor, with the most common location being the left atrium. Atrial myxomas are associated with three major complications including obstruction, emboli, and constitutional symptoms such as fever and weight loss [3]. These symptoms may be challenging to identify especially in the presence of other illnesses which highlights the value of reviewing the clinical presentation of atrial myxomas.

## Case presentation

A 54-year-old morbidly obese Caucasian female with a past medical history of hypothyroidism, type 2 diabetes mellitus, and tobacco abuse presented to the Emergency Department with excruciating back pain and shortness of breath (SOB). Four days prior, she had been evaluated at her local clinic for SOB, back pain, nausea and vomiting where she received a diagnosis of pneumonia and a course of antibiotics, Rocephin and Azithromycin. The patient's vitals upon arrival to the Emergency Department can be seen in Table 1.

**Table 1:** Vitals upon arrival at the Emergency Department

Vitals (reference range)	
Temperature	102.4° F (97.8-99.1° F)
Heart rate	101 Beats per minute (BPM) (60-100 BPM)
Respiratory Rate	22 breaths per minute (12-18 breaths per minute)
Blood Pressure	127/72 mmHg (90/60 mm Hg to 120/80 mm Hg)
Pulse oximetry	96% on room air (<95% or higher)

Physical examination showed a morbidly obese female in moderate discomfort. Lungs were clear auscultation to bilaterally without wheezing present. The cardiac exam showed regular rate and rhythm with no murmurs appreciated. There was no Jugular Venous Distention (JVD) present and pulses were palpable to bilateral upper and lower extremities. No edema was noted in the bilateral lower extremities. The abdomen was soft, non-tender, and non-distended with normoactive bowel sounds in all four quadrants. Electrocardiogram showed sinus tachycardia with no acute ST changes. The patient’s labs taken in the Emergency Department can be seen in Table 2.

**Table 2:** Laboratory Values from the Emergency Department

Emergency Department Lab Values (reference range)	
WBC	12,000/mcL (5,000-10,000/mcL)
Creatinine	1.1 mg/dL (0.6-1.2 mg/dL)
BNP	3140 (<100 pg/mL)
d-dimer	Elevated

**Figure 1:**
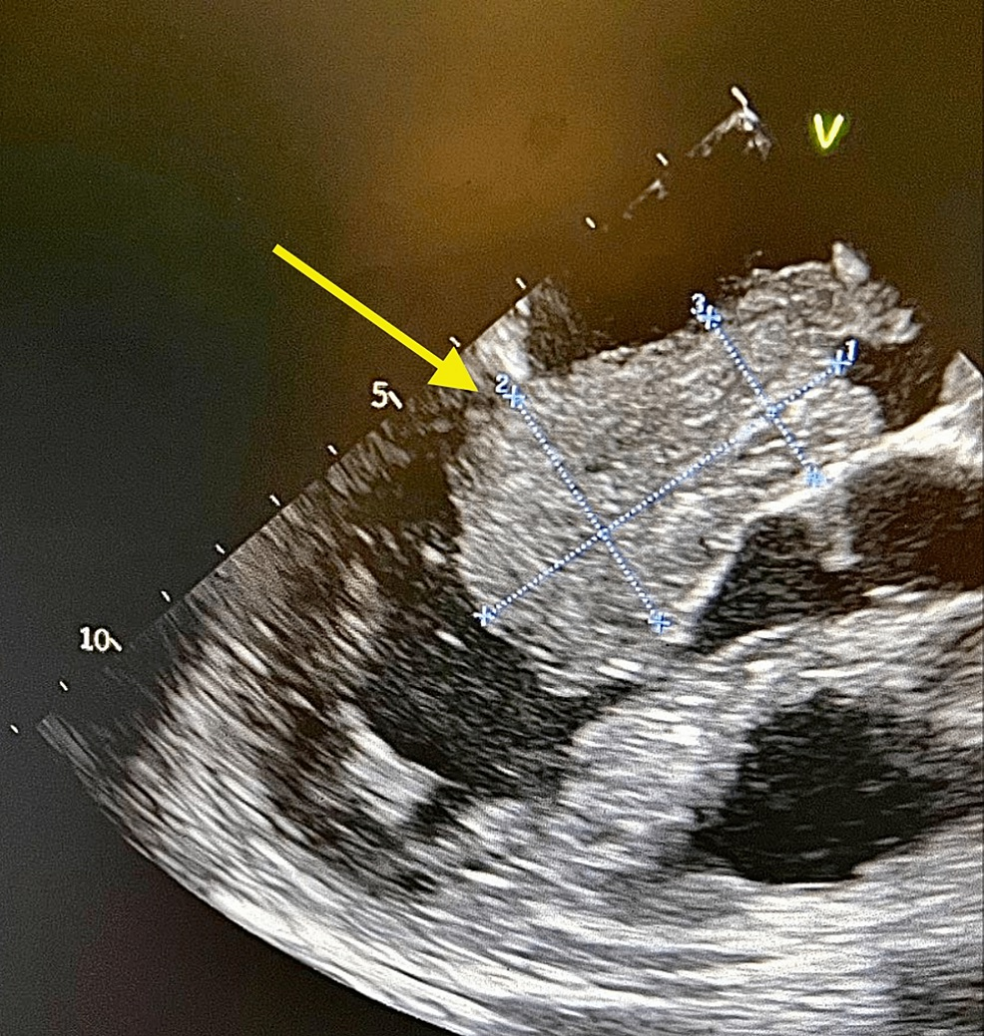
Left atrial myxoma is shown by the yellow arrow. The measured dimensions of the mass in the echocardiogram were 4.9 cm, 3.1 cm, and 2.1 cm indicated by lines 1, 2, and 3, respectively.

Cardiac enzymes were unremarkable. Computed topography (CT) angiography of the chest showed no pulmonary embolus. The elevation of brain natriuretic peptide (BNP) was unusual given the patient had no prior history of heart failure. In order to rule out congestive heart failure, an echocardiogram was ordered. Imaging showed an ejection fraction of 50%-55%, mild left atrial enlargement with large pedunculated, mobile mass attached to left atrial body which was moving through the mitral valve during diastole (Figure 1). The mass measured to be 6.2 x 2.7 cm per transesophageal echo (TEE) report.

**Figure 2:**
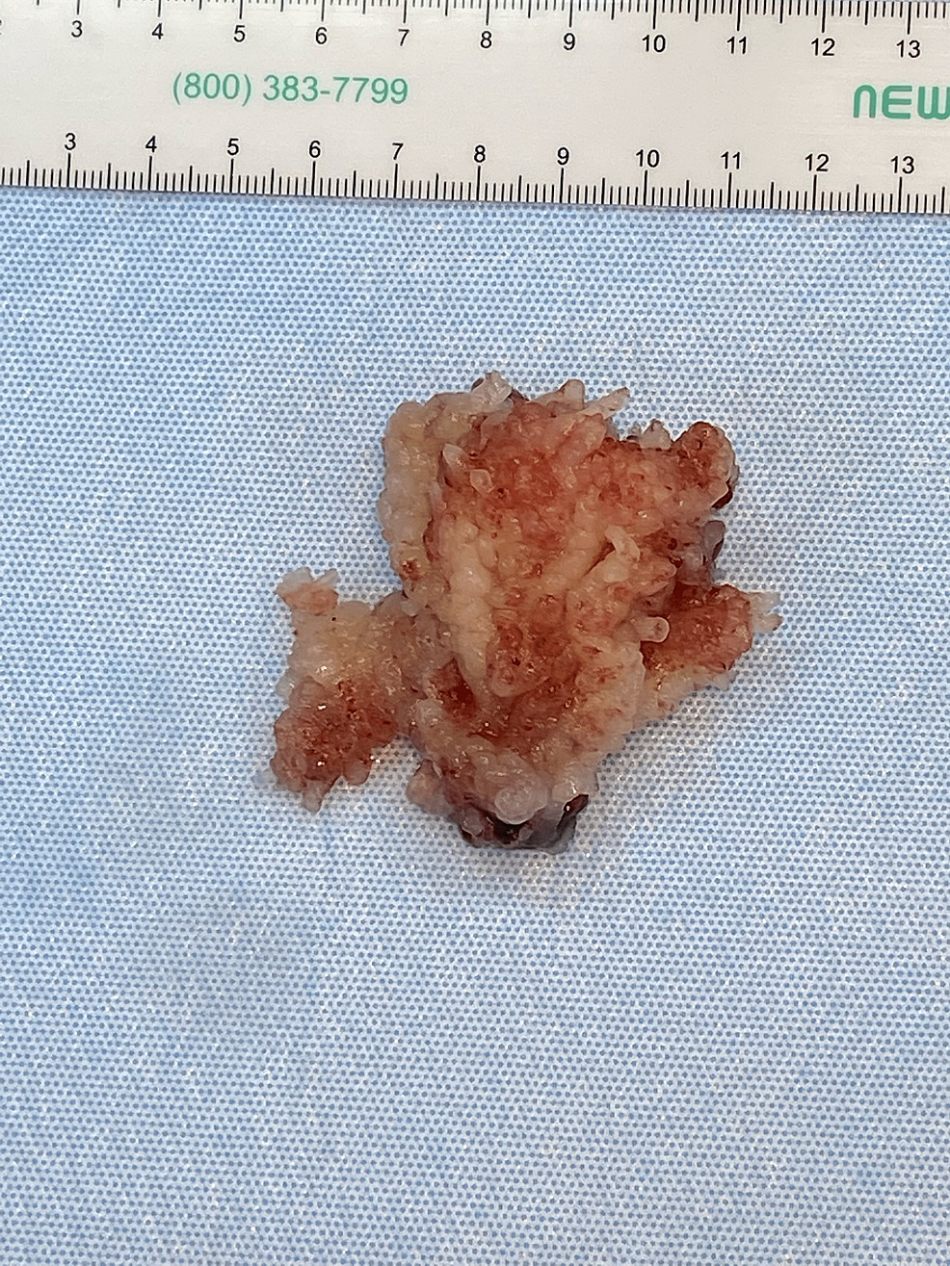
Left atrial myxoma measuring 6.2 cm x 2.7 cm

A diagnosis of left atrial myxoma was made and the Cardiothoracic Surgeon was consulted for surgical intervention. A cardiac catheterization was completed to delineate the coronary anatomy and ensure the cardiovascular system would be able to undergo the surgery. Once cleared, the patient was taken to surgery. The left atrium was opened over the top of the right superior pulmonary vein and the myxoma was easily identified with a stalk off of the septum. The myxoma was removed by holding the stalk and pulling it out. The myxoma can be seen in Figure 2.

The operation was successful without any complications and the patient was taken to recovery. The patient was observed in the hospital for five days and was discharged home with anticoagulation therapy and plan to follow up in office in 14 days. Following her discharge, her symptoms had resolved.

## Discussion

Atrial myxomas have a discrete presentation which may pose challenges to their diagnosis during clinical workup. The wide range of differential diagnoses of patients presenting with back pain are SOB such as the patient presented are vast, and unfortunately, there are more common diagnoses that are used prior to an atrial myxoma diagnosis. The concern with this patient, as discussed above, was the elevated BNP without the presence of congestive heart failure symptoms such as peripheral edema or arrhythmias. The SOB that was present was thought to be secondary to her recent pneumonia diagnosis four days prior. Without the echocardiogram, which was completed due to an increase in BNP, it is possible that the atrial myxoma would have possibly gone unnoticed. Furthermore, upon physical examination there were no distinct heart sounds noticed which could have increased suspicion of a cardiac tumor.

Clinical symptoms of atrial myxoma are mostly dependent on the size of the tumor, as it can obstruct blood flow from different chambers of the heart. In most cases, the myxoma is found in the left atrium, similar to our patient in the case. The pedunculated mass can block blood flow from the left atrium to the left ventricle, causing there to be a backflow of blood [4]. This can explain the SOB the patient experienced as there is excess blood that becomes pooled in the pulmonary vasculature, mimicking the symptoms of left ventricular heart failure.

Other symptoms which may arise from atrial myxoma may include thromboembolic, constitutional, and those related to Carney’s complex, a rare multiple endocrine neoplasia syndrome [5]. The left atrium is an area with high systolic pressure which make it prone to an increased chance of systemic embolization, especially in the central nervous system. In the event of an embolization, Patients may present with a transient ischemic accident, loss of vision, or hemiplegia. Furthermore, patients may present with fever, weight loss, and malaise due to the release of cytokines (ex IL-6) which play a role in the proliferation of myxoma cells [6]. The fever that our patient presented with was originally attributed to the prior pneumonia diagnosis rather than the myxoma. Lastly, this cardiac tumor is associated with Carney’s complex which presents as unusual skin pigmentation, develops tumors in endocrine tissues, and will have recurrent atrial myxomas.

When approaching a differential diagnosis of atrial myxoma, understanding the wide range of symptoms is paramount. Dyspnea is the most common symptom associated with myxomas secondary to the impeded blood flow from the heart. Other respiratory symptoms can include orthopnea, paroxysmal nocturnal dyspnea, pulmonary edema, and cough. Peripheral edema is another common symptom secondary to decreased peripheral blood flow and early signs of heart failure. Constitutional symptoms can include weight loss, fever, dizziness, and fatigue secondary to decreased blood flow.

On physical examination, there are several things to watch out for. On cardiovascular examination, there may be heart sounds that are similar to mitral stenosis may be audible including a loud S1. A tumor plop sound is distinguished as an early diastolic sound secondary to the tumor hitting the endocardial walls following contraction. If there is a tumor obstructing a valve, a characteristic rumbling sound may be heard at the associated listening post of the valve involved. S3 and S4 may also be audible. Jugular venous pressure may be elevated with an associated delay of P2 if pulmonary hypertension is present. Any combination of these exam findings when paired with the patient’s presentation should move a cardiac myxoma up the differential diagnosis list.

Should further evaluation be indicated, it is imperative to order imaging to help determine the location, size, and severity of the myxoma. The most effective imaging study is echocardiography. There are two ways in which the echocardiogram can be obtained; transthoracic or transesophageal. A transthoracic echocardiogram (TTE) is simple and highly effective but can be obscured by bone and other structures in the thoracic cavity. Transesophageal echocardiography, while more invasive, decreases the amount of intervening structures simply due to the anatomical proximity of the esophagus to the heart. It has been shown to have 100% sensitivity and a high specificity than transthoracic echocardiography [7]. Other options for non-invasive imaging include cardiac magnetic resonance imaging and computed tomography, positron emission tomography (PET) scan, and coronary angiography. If a myxoma is detected prompt surgical consultation is imperative. Removal of the myxoma is highly indicated based on the risk of cardiac complications including potential embolization.

Surgical intervention is one of the only treatments that are available for cardiac myxomas and is indicated secondary to potential complications that can occur in the presence of the tumor. Median sternotomy is the most commonly used surgical technique for myxoma excision with some recent reports of cases utilizing a robotic approach [8]. A wide resection of the tumor base is imperative to avoid recurrence along with careful inspection of surrounding cardiac tissue [9]. The recurrence rate of myxomas is about 3% [7]. Most patients tolerate the surgery well with an average recovery time of about 8 weeks post median sternotomy. Long-term management includes clinical evaluation, as well as echocardiography, and follow up to monitor all cardiac chambers [10].

## Conclusions

Successfully diagnosing atrial myxoma is paramount to improve patient prognosis, however, the clinical presentation makes it challenging. The symptoms are ones that have many differential diagnoses such as back pain and SOB as our patient presented, both of which can be attributed to different diseases. In this case the SOB was attributed to the prior diagnoses of pneumonia. Appropriate lab values and associated imaging for any outliers, such as the elevated BNP, are highly valuable in making the diagnosis of atrial myxomas. Echocardiograms are highly sensitive to atrial myxomas and should be utilized for diagnosis and identification of location. However, there needs to be clinical indication to order the imaging of the heart, which further emphasizes the importance of case reviews such as this to educate on possible presentations of atrial myxomas to ensure accurate diagnostic practices and effective patient care.
